# Localized delivery of therapeutic doxorubicin dose across the canine blood–brain barrier with hyperthermia and temperature sensitive liposomes

**DOI:** 10.1080/10717544.2018.1461280

**Published:** 2018-04-24

**Authors:** Amy Lee Bredlau, Anjan Motamarry, Chao Chen, M. A. McCrackin, Kris Helke, Kent E. Armeson, Katrina Bynum, Ann-Marie Broome, Dieter Haemmerich

**Affiliations:** a Department of Neuroscience, Medical University of South Carolina, Charleston, SC, USA;; b Regeneron Pharmaceuticals, Inc., Tarrytown, NY, USA;; c Department of Cell and Molecular Pharmacology & Experimental Therapeutics, Medical University of South Carolina, Charleston, SC, USA;; d Department of Pediatrics, Medical University of South Carolina, Charleston, SC, USA;; e Department of Drug Discovery and Biomedical Sciences, Medical University of South Carolina, Charleston, SC, USA;; f Department of Comparative Medicine, Medical University of South Carolina, Charleston, SC, USA;; g Department of Public Health Sciences, Medical University of South Carolina, Charleston, SC, USA;; h College of Charleston, Charleston, SC, USA

**Keywords:** Thermosensitive liposomes, thermal therapy, blood–brain barrier, hyperthermia, doxorubicin, liposomes

## Abstract

Most drugs cannot penetrate the blood–brain barrier (BBB), greatly limiting the use of anti-cancer agents for brain cancer therapy. Temperature sensitive liposomes (TSL) are nanoparticles that rapidly release the contained drug in response to hyperthermia (>40 °C). Since hyperthermia also transiently opens the BBB, we hypothesized that localized hyperthermia can achieve drug delivery across the BBB when combined with TSL. TSL-encapsulated doxorubicin (TSL-Dox) was infused intravenously over 30 min at a dose of 0.94 mg/kg in anesthetized beagles (age ∼17 months). Following, a hyperthermia probe was placed 5–10 mm deep through one of four 3-mm skull burr holes. Hyperthermia was performed randomized for 15 or 30 min, at either 45 or 50 °C. Blood was drawn every 30 min to measure TSL-Dox pharmacokinetics. Nonsurvival studies were performed in four dogs, where brain tissue at the hyperthermia location was extracted following treatment to quantify doxorubicin uptake via high-performance liquid chromatography (HPLC) and to visualize cellular uptake via fluorescence microscopy. Survival studies for 6 weeks were performed in five dogs treated by a single hyperthermia application. Local doxorubicin delivery correlated with hyperthermia duration and ranged from 0.11 to 0.74 μg/g of brain tissue at the hyperthermia locations, with undetectable drug uptake in unheated tissue. Fluorescence microscopy demonstrated doxorubicin delivery across the BBB. Histopathology in Haematoxylin & Eosin (H&E) stained samples demonstrated localized damage near the probe. No animals in the survival group demonstrated significant neurological deficits. This study demonstrates that localized doxorubicin delivery to the brain can be facilitated by TSL-Dox with localized hyperthermia with no significant neurological deficits.

## Introduction

High-grade gliomas (brain tumors such as glioblastoma and diffuse astrocytoma) are associated with poor survival and limited effective treatment options. Such gliomas are expected to occur in 26,070 people in the USA in 2017, with poor survival rates; 5- and 10-year survival is 34.9% and 29.3%, respectively (CBTRUS, 2017). A major limiting factor for drug-based therapies is the blood brain–barrier (BBB), which prevents or limits drug uptake by brain tissue (Groothuis, 2000; Lesniak & Brem, 2004; van Tellingen et al., 2015). While the BBB is often compromised in some brain tumor regions, there are usually tumor regions with intact BBB, and thus inaccessible by drugs (van Tellingen et al., 2015). Clearly, strategies that can circumvent the BBB would be beneficial to the treatment of aggressive brain tumors, especially if they could be implemented in a localized fashion, thereby reducing CNS toxicity of the therapeutic agents against healthy brain tissue or toxicity to nontarget organs. Strategies to open or circumvent the BBB include convection-enhanced delivery (where drug is infused directly into the interstitial space), hyperosmolar BBB disruption, and focused ultrasound combined with microbubbles, among others (Groothuis, 2000; Carpentier et al., 2016). In addition, hyperthermia (>41 °C) has been demonstrated to transiently open the BBB in animals and human patients (Ikeda et al., 1994; Kakinuma et al., 1996; Kiyatkin & Sharma, 2009; Leuthardt et al., 2016).

Temperature sensitive liposomes (TSL) are drug delivery systems that release the encapsulated drug in response to mild hyperthermia, typically above ∼40 °C (Gaber et al., 1995; Needham et al., 2000; Lindner et al., 2004; Motamarry et al., 2017). The first study investigating the use of hyperthermia and temperature sensitive liposomes (TSL) for drug delivery to the brain was performed in the 1990s with earlier TSL formulations, which showed that the chemotherapy agent cisplatin can be delivered across the BBB using TSL in combination with hyperthermia in healthy rats. The same study further demonstrated delivery of Evans blue dye from TSL across the BBB in normal dog brain, in regions exposed to temperatures >41 °C (Kakinuma et al., 1996). The same authors followed up with a rodent model of glioblastoma for delivering cisplatin to the tumor with TSL and hyperthermia (Kakinuma et al., 1996). Later studies showed that localized hyperthermia combined with intra-arterial infusion of doxorubicin could deliver doxorubicin to glioma in rats, resulting in improved treatment outcome (Morita et al., 2003a), and that use of doxorubicin-TSL instead of drug infusion further improved drug uptake (Aoki et al., 2004; Gong et al., 2011). However, since the BBB was supposedly compromised in these tumor models, these studies did not specifically demonstrate delivery of doxorubicin across the BBB. Prior studies examining the impact of hyperthermia on delivery of nonthermosensitive liposomes found no impact on normal brain delivery (Wu et al., 2014, 2017). While hyperthermia itself may enhance delivery of free drug due to enhanced cell uptake (Osborne & MacKillop, 1987), one prior study demonstrated limited increase from 0.19 to 0.26 μg/g of Dox in rat brain tumors (Morita et al., 2003b), and another study with free cisplatin in normal rat brain found no impact of hyperthermia (Kakinuma et al., 1996). One study combined either free Dox or Doxil with whole-brain hyperthermia and found limited drug uptake for both (0.1–0.2 μg/g) (Gong et al., 2011). Furthermore, there have not been any studies demonstrating TSL-based drug delivery across the BBB in large animals, and none of the studies above employed the newer, rapid-release TSL formulations that release their content within seconds (Needham et al., 2000; Lindner et al., 2004). These newer formulations are based on the intravascular triggered delivery concept, where drug is released from TSL within the capillaries and then taken up by tissue (Gasselhuber et al., 2012; Manzoor et al., 2012; Kneidl et al., 2014; Motamarry et al., 2017).

**Figure 1.**
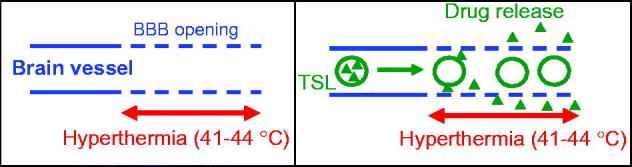
Intended drug delivery paradigm: (A) BBB is locally opened (indicated by dashed vessel walls) in the region where hyperthermia is applied. (B) Drug (*triangles*) is released from TSL (*circles*) in region where BBB is opened, facilitating localized drug delivery across the BBB.

We hypothesized that the combination of hyperthermia with these newer, fast-release TSL formulations may be effective in delivering drugs across the BBB. In this paradigm, hyperthermia opens the BBB, and at the same time triggers intravascular release from TSL in the tissue regions where the BBB has been compromised, with subsequent tissue uptake (Figure 1). We selected doxorubicin as our therapeutic agent. Doxorubicin is currently not used in the treatment of brain tumors because it cannot cross the BBB, but is effective against high-grade gliomas in culture (Stan et al., 1999; Veringa et al., 2013; Sewing et al., 2017) and *in vivo* (Gong et al., 2011; Sewing et al., 2017), and also patient benefit has been demonstrated when administered in liposomal form (Fabel et al., 2001). Thus, proof of doxorubicin delivery across the BBB has potential therapeutic value, but would also demonstrate viability of the proposed approach in delivering drugs across the BBB that otherwise could not cross (Sewing et al., 2017). In addition, we wanted to examine long-term survival in large animals to evaluate the neurotoxicity of doxorubicin delivered to focal areas of the brain in the setting of transient hyperthermia, which has not been evaluated in prior studies.

There are a number of eligible brain models for investigation of therapies for high-grade gliomas. Mice are often used, as they can be bred for multiple purposes. However, their brains are approximately the size of a lima bean, and so hyperthermia is hard to keep localized in such tight quarters. Larger mammals are superior for the assessment of both efficacy and toxicity of localized hyperthermia and allow for the use of hyperthermia probes of same size as used in human patients rather than customized miniature probes for rodent use. Canines are particularly useful as models for brain tumors as they develop *de novo* tumors that are similar to those of humans (e.g. glioblastomas and meningiomas), so they are useful models for both proof of principle and efficacy trials. Hence, for this study, a canine model was used. Notably, research results are relevant to the treatment of brain tumors in both humans and companion dogs.

## Materials and methods

### Preparation of temperature sensitive liposomes encapsulating doxorubicin

The TSL encapsulated doxorubicin (TSL-dox) was prepared according to Needham et al. (2000) with modified lipid composition as described in a prior study (Saxena et al., 2015). The lipids 1,2-dipalmitoyl-sn-glycero-3-phosphocholine (DPPC), 1,2-distearoyl-sn-glycero-3-phosphoethanolamine-N-PEG2000 (DSPEPEG2000), and monostearoylphosphatidylcholine (MSPC) (Avanti Lipids, Alabaster, AL) were dissolved in chloroform and dried under a stream of atmospheric air at room temperature for forming a thin film. The lipid ratio was DPPC:MSPC:DSPE-PEG2000 (85.3:9.7:5.0 mol%). The lipids were then hydrated with 300 mM citric acid buffer (pH 4.0) and extruded at 55 °C (Thermobarrel extruder; Northern Lipids, BC, Canada) through 100 nm filter. Active loading of doxorubicin into the liposomes was carried out by pH gradient with phosphate buffered saline (PBS, pH 7.4) outside the liposomes. The liposome size was measured by dynamic light scattering (ZetaPALS, Brookhaven, CT) with 20 µl of liposomes diluted in 3 ml of PBS.

The release kinetics of the doxorubicin from the TSL was measured between 37 and 45 °C by a millifluidic device as described earlier (Burke et al., 2017). Briefly, TSL-dox was diluted to 80 µg/g concentration in PBS at room temperature (∼25 °C). The solution carrying the liposomes was pumped through a glass capillary tube (flow rate ∼50 µl/min), with the tube pre-heated to the desired temperature by a heating element (Burke et al., 2017). Since doxorubicin fluorescence is quenched while encapsulated, drug release causes a fluorescence increase. As the solution – initially at room temperature – enters the heated capillary, drug release from TSL can be measured as a fluorescence gradient along the tube length (Burke et al., 2017). The fluorescence intensity of unencapsulated doxorubicin was considered as corresponding to 100% release. Fluorescent imaging of the tube was performed with an imaging system (Maestro; Caliper Life Sciences, Hopkinton, MA), and the images were processed using ImageJ software (NIH, MD) to determine release kinetics at varying temperatures. To quantify TSL stability at body temperature, the TSL solution inside the capillary tube was maintained at 37 °C for 20 min without pumping.

### Hyperthermia system

We employed a commercial radiofrequency generator (Cool-tip RF generator; Medtronic, Boulder, CA) and 17-gauge radiofrequency ablation probe (Cool-tip needle electrode; Medtronic, Boulder, CA) to facilitate localized hyperthermia. The probe has an integrated temperature sensor; during hyperthermia, power was manually adjusted to keep the temperature measured by this sensor at either 45 or 50 °C (Table 1). Probe temperature was recorded in 3 s intervals.

**Table 1.** Time averaged temperature over hyperthermia duration (15 or 30 min), shown for both nonsurvival studies (four hyperthermia treatments per dog) and survival study (one hyperthermia treatment per dog).

Probe number	Dog 1	Dog 2	Dog 3	Dog 4	
1	45.2 ± 0.1 (15 min)	45.2 ± 0.2 (30 min)	50.1 ± 0.3 (15 min)	50.1 ± 0.1 (30 min)	–
2	45.1 ± 0.4 (30 min)	(45)^a^ (15 min)	50.3 ± 0.2 (30 min)	50.0 ± 0.2 (15 min)	–
3	50.3 ± 0.4 (15 min)	50.2 ± 0.2 (15 min)	45.2 ± 0.3 (15 min)	45.1 ± 0.1 (30 min)	–
4	50.2 ± 0.2 (30 min)	50.2 ± 0.3 (30 min)	45.2 ± 0.1 (30 min)	45.0 ± 0.1 (15 min)	–
Survival dog number	1	2	3	4	5
	(45)^a^ (30 min)	49.9 ± 0.4 (30 min)	50.0 ± 0.3 (30 min)	50.0 ± 0.3 (30 min)	49.9 ± 0.2 (30 min)

Temperatures are shown as average (°C) ± SD; duration is listed in brackets.

a In two cases, temperatures were not recorded due to malfunction of the recording system, and the intended target temperature is listed in brackets. However, since temperature was controlled in same fashion, we can assume similar accuracy as listed for the other hyperthermia treatments.

In preliminary *in vitro* studies, we measured the temperature profile induced by the hyperthermia probe in tissue mimicking gel, similar to a prior study (Deshazer et al., 2017). Briefly, we placed the probe on top of a cylindrical Agar gel phantom (3% Agar, 0.25% NaCl). Generator power was manually adjusted to keep probe tip temperature at either 45 or 50 °C. During heating, the gel surface temperature was captured with an infrared camera (Microspec 7500; LumaSense Tech., Santa Clara, CA) at a frame rate of 1/s.

### Animal oversight and husbandry

Animal activities were approved by the MUSC IACUC, which provides oversight for the AAALAC-accredited animal research program. Intact adult Beagles of both sexes were procured from a Class A USDA-licensed commercial dog breeder (Covance Research Products, Inc., Cumberland, VA) and acclimatized a minimum of five days before nonsurvival and 7 days before survival surgery. Housing included 12:12 light:dark cycle, *ad lib* reverse osmosis water, and twice daily feeding (Pro Plan Focus Sensitive Skin and Stomach; Nestle Purina Pet Food Co., St. Louis, MO). Dogs scheduled for survival surgery were bathed with chlorhexidine soap 1–4 d before surgery.

### Anesthesia and analgesia

All dogs were assessed by physical examination, complete blood count, and clinical chemistry. They were treated with oral antihistamines (famotidine, 0.5 mg/kg once daily; diphenhydramine, 2 mg/kg TID) and steroids (prednisone, 0.5 mg/kg BID) for 24 h before nonsurvival and 2 d before and 1 d after survival surgery to prevent allergic reactions to the liposomes. On the day of surgery, dogs were pre-anesthetized intramuscular (IM) with midazolam (0.25 mg/kg) and buprenorphine (0.05 mg/kg), a 22-gauge intravenous (IV) cephalic catheter was placed, and anesthesia was induced IV with propofol (4.7 mg/kg, to effect). Dogs were intubated and maintained under anesthesia with isoflurane in 100% O_2_ using a mechanical ventilator. Supportive care included lactated ringers solution (LRS) (10 ml/kg/h IV) through the cephalic catheter, external thermal support, and perioperative antibiotics (survival surgery only; cefazolin 22 mg/kg IV 30 min before incision and every 90 min thereafter until incision closure). The external thermal support was adjusted to keep the body temperature below 37 °C to limit systemic leakage of doxorubicin from TSL. A 19 gauge, 8-inch catheter (Intracath; Argon Medical, Athens, TX) was placed percutaneously into the right jugular vein for serial sample collections for pharmacokinetics (PK) analysis. Bupivacaine 0.5% (1 mg/4.5 kg) was infused subcutaneously (SC) along the dorsal midline of the head where the skin incision was planned. Additional antihistamines (famotidine, 0.5 mg/kg; diphenhydramine, 2 mg/kg) and steroids (dexamethasone, 2 mg/kg) were given IV before TSL-dox infusion.

### Nonsurvival surgery

**Figure 2.**
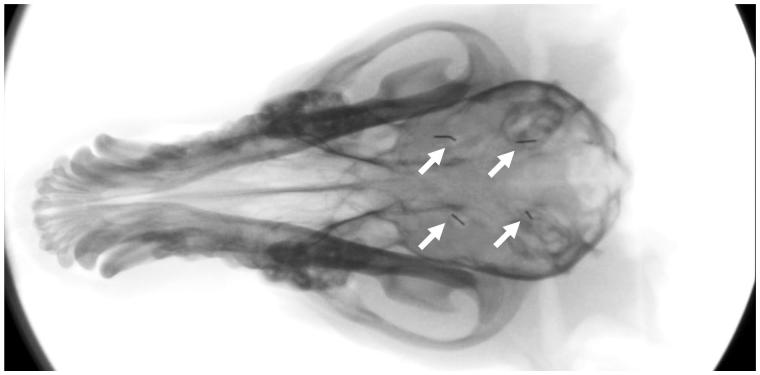
The four locations where hyperthermia was applied are indicated by fiducial markers (white arrows) in this X-ray image. The hyperthermia probe was placed through burr holes, penetrating ∼10–15 mm deep into brain tissue.

A dorsal midline approach to the skull was made in four dogs (two males, two females) averaging 8.2 kg (range: 7.8–8.4; SD: 0.3) and 16.9 months old (range: 16.2–17.5; SD: 0.6) and the temporal muscle fascia incised and the muscle elevated bilaterally. Four 3-mm trephinations were created with a high-speed nitrogen-driven surgical drill (Hall Surgairtome II; ConMed Linvatec, Largo, FL), marking the corners of a square with sides 2 cm in length. Each trephination was located 1 cm lateral to midline. After trephinations were made, dogs were treated with IV TSL-dox infusion over 30 min. A hyperthermia probe was then placed 5–10 mm deep into the brain and hyperthermia treatments administered in random order in four different areas of the brain (Figure 2), for 15 or 30 min at 45 or 50 °C. A factorial design was used to obtain one measurement per animal for each temperature and time duration level. Order of treatment conditions (time, temperature) was specified to minimize bias in the timing and administration of TSL-dox. Blood collection for PK analysis was done at baseline, immediately after completion of TSL-dox infusion, and at 30, 60, 90, 120, 150, and 180 min after TSL-dox infusion was complete. Blood was drawn from the jugular catheter dedicated to sample collections to avoid contamination from TSL-dox administration, which was given IV through the cephalic catheter. After completion of hyperthermia in all four areas of the brain, each canine was euthanized with pentobarbital (86 mg/kg) IV, and the brain was collected for assessment of the delivery of doxorubicin as well as determination of cellular damage due to the procedure.

### Survival surgery

Five dogs (three males, two females) averaging 7.7 kg (range: 7.4–8.2; SD: 0.3) and 9.3 months of age (range: 8.75–9.75; SD: 0.4) were treated with IV TSL-dox and a single localized dose of hyperthermia using a left rostrotentorial approach. The first dog was treated at a more conservative hyperthermia protocol of 45 °C for 30 min. Following successful recovery without neurological deficits, the remaining four dogs were treated at the most aggressive protocol that resulted in the largest drug delivery, 50 °C for 30 min. Blood collection for PK analysis was done at baseline, immediately after completion of TSL-dox infusion, and a third time 40–60 min later. Dogs were recovered from anesthesia and were monitored for neurologic or hematologic toxicities for 6 weeks prior to euthanasia and assessment of the brain tissue. Dogs received detailed clinical neurological assessments 1–4 days preoperatively, 3–4 days postoperatively, and before euthanasia (39–42 days postoperatively). They were assessed daily for general health and gait abnormalities. Their progress was also followed with weekly complete blood counts done to monitor for hematologic toxicities after single administration of TSL-dox.

### Pathology and histology

For each probe placement in dogs undergoing nonsurvival surgery, a 2 × 2 cm cube was dissected with the probe placement area in the center of the section for each of four probe placements. Sections were subsequently bisected coronally with the probe placement in the center. The caudal hemisection was saved for drug quantification via high-performance liquid chromatography (HPLC) and the cranial section was further dissected into lateral and medial quadrants for microscopic analysis. The medial quadrants were immersion fixed in formalin and trimmed transversely for Haematoxylin & Eosin (H&E) staining and histologic analysis, and lateral quadrants were embedded in optimal cutting temperature (OCT) compound and frozen at –80 °C. Formalin fixed tissues were sectioned transversely into dorsal, middle and ventral sections, embedded in paraffin and 5 um sections placed on negatively charged glass slides for analysis. Frozen sections were sectioned transversely and stained with Hoechst 33342. Dorsal, middle, and ventral aspects of the lesioned brain area were examined separately under florescent excitation at 360 nm and emission at 460 nm (for detection of cell nuclei via DAPI) and excitation and emission at 540 nm and 605 nm (for doxorubicin detection) (Shen et al., 2008).

For each animal that was followed 6 weeks postoperatively, the brain was removed and bisected along the midline, and immersion fixed in 10% of neutral buffered formalin. In these animals, only one probe placement was present. Both coronal and transverse sections centered on the gross lesion were examined histologically. A coronal section was made at the level of the lesion and one coronal section was examined for histology cranial to the lesion. For the section caudal to the lesion, transverse sections at 1–2 mm intervals were placed into cassettes and histology examined.

### HPLC quantification of doxorubicin in plasma and tissue

After sectioning of the brain, 15–105 mg of tissue was selected from the brain area around which the hyperthermia probe was inserted. For each tissue piece, three samples were extracted for quantification: (1) within 2 mm of the probe, (2) 2–4 mm from the probe, and (3) at >4 mm distance (control samples). This tissue was homogenized, combined with acetone for cell lysis and chloroform for extraction of doxorubicin and idarubicin (added as an internal standard) from the tissue sample. For plasma samples, 100 μl of plasma was used and processed as above, without homogenization. Chloroform extraction was repeated four times prior to doxorubicin and idarubicin extraction and resuspension in HPLC running solution. This was centrifuged and the supernatant was analyzed via HPLC.

Each tissue sample (15–105 mg) was doped with idarubicin (Ida, as internal standard of 0.25 μg/ml in the prepared HPLC sample) and then homogenized and lysed in the Kontes 2 mL All Glass Dounce Tissue Grinder. The lysis agent was acetone. With acetone for lysis, 200 μl of a pH 9 buffer (0.9 M sodium carbonate and 0.1 M sodium bicarbonate) was filled, followed by grinding for homogenization; afterward, 200 μl of acetone was added and the mixture was agitated for lysis using the grinder pestle. After lysis, 1 ml of chloroform was added with agitation to extract Dox and Ida. The organic phase was layered out by centrifugation and collected for chloroform evaporation under vacuum using a Büchi Rotavapor R-210 (BUCHI corp., New Castle, DE). The extraction was repeated four times for one lysed sample and the extracted materials were collected together.

At room temperature (∼23 °C), 50 μl of sample was injected into the reverse phase column and eluted with the mobile phase (running solution) of 30% of acetonitrile plus 70% of a pH 2.5 buffer (91.4 mM citric acid and 8.6 mM sodium phosphate dibasic). The elution rate was 1 ml/min. The elution profile was monitored using fluorescence detection at 558 nm (480 nm excitation). The flow cell temperature was 45 °C.

### Fluorometry quantification of doxorubicin in plasma

Plasma samples of both acute and survival animals were analyzed by fluorometry. While this method is not sufficiently sensitive for tissue samples, it works adequately in plasma samples and is more cost-effective than HPLC. Plasma samples were collected and stored at –80 °C. Doxorubicin concentration was quantified as previously described (Brouckaert et al., 2004), with slight modifications. Briefly, the samples were thawed on ice. To 30 μl of plasma sample, 90 μl of PBS and 100 μl of 10% of Triton ×100 (diluted in distilled water) were added to release liposomal doxorubicin. Sample fluorescence was measured via fluorescence microplate reader (Synergy HT, Biotek Instruments Inc., Winooski, VT) with appropriate excitation and emission filters for doxorubicin (excitation 485 nm; emission 590 nm). The drug concentration was calculated from fluorescence based on a standard curve prepared from dog plasma samples spiked with known concentrations of doxorubicin.

### Statistical analysis

Sixteen drug concentration results were analyzed using a linear mixed model with temperature and duration as fixed effects, and a random factor on the animal to account for within-animal correlation (Model A). Least square mean estimates were calculated for each of the four temperature and duration combinations. The temperature, duration interaction was tested but ultimately removed from the final model as the *p* value for the interaction was .74, and least square estimates with the interaction term included were very similar to those without the interaction (differences in estimated concentration <0.02 in each case). To identify which variable would be a better predictor of concentration levels, a second mixed model (Model B) was used with the continuous variable of area under the curve (AUC) replacing duration AUC was calculated from plasma PK data (Figure 5(A)) during the duration when hyperthermia was applied. Again, the interaction term was not included in the final model (*p* = .96). The negative log-likelihood was calculated for each of the two models to assess fit, and a likelihood ratio test was used to test if the two models are significantly different. Finally, we removed the random components from the models to compare the simple *R*^2^ values between the two models. We set *α* = 0.05 for all tests for statistical significance.

## Results

### In vitro characterization of temperature sensitive liposomes

**Figure 3.**
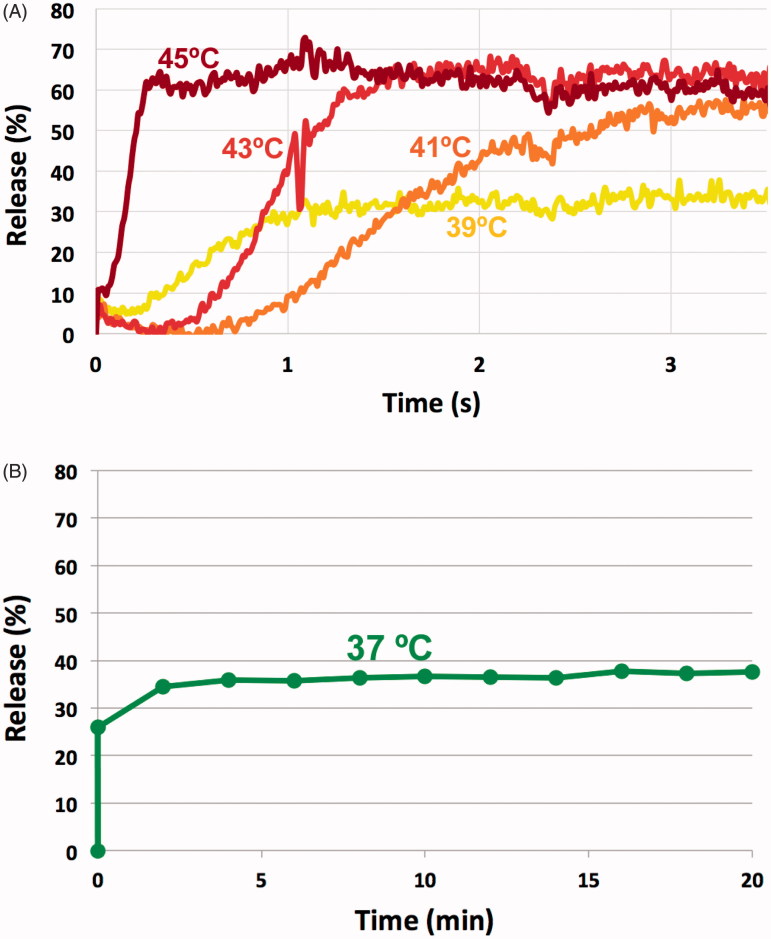
TSL release kinetics. (A) Doxorubicin is almost completely released within ∼2 s when TSL are heated to above 41 °C. (B) TSL stability at body temperature: Approximately 35% of drug is released at 37 °C within the first 5 min, but remaining drug remains stable within TSL.

The liposome size was 111 ± 5.3 nm. Optimal release was achieved at temperatures above 41 °C, with release of 90–100% of the drug within <2 s (Figure 3). Release was negligible at room temperature (data not shown). About 25% of drug was release within a few seconds after heating to 37 °C, followed by slow leakage. Note that the time scale in Figure 3(B) is in minutes to examine leakage within the systemic plasma during longer duration, compared to seconds time scale in Figure 3(A).

### Hyperthermia probe

**Figure 4.**
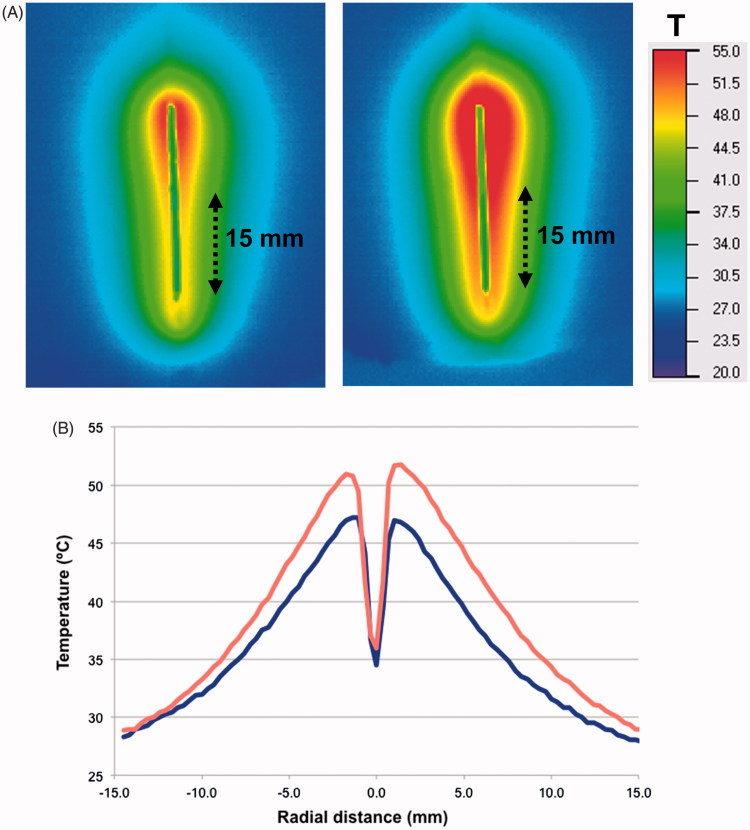
Hyperthermia probe temperature maps measured *in vitro* in gel phantoms. (A) Temperature maps after 3 min heating are plotted at 45 (left) and 50 °C (right) [probe tip at bottom; in animal studies, only the distal 15 mm was inserted into tissue (arrows)]. (B) Radial temperature profile ∼7 mm from the probe tip for 45 and 50 °C. Temperatures exceeding 41 °C (i.e. where doxorubicin release from TSL is expected) extend ∼4 and ∼6 mm from the probe for 45 and 50 °C, respectively. *In vivo*, these distances are likely somewhat reduced since blood perfusion-mediated cooling limits temperature spread into tissue.

Figure 4 shows temperature data resulting from heating of gel phantoms at 45 and 50 °C probe temperature. Hyperthermia was applied until approximate steady-state conditions were achieved (∼3 min). Note that during hyperthermia *in vivo*, blood perfusion mediated cooling will result in somewhat lower temperatures than observed in these *in vitro* studies. Further, we only inserted the probe to ∼10–15 mm depth in dogs, whereas the whole 30 mm was in contact with the gel phantom.

### Plasma doxorubicin pharmacokinetics

**Figure 5.**
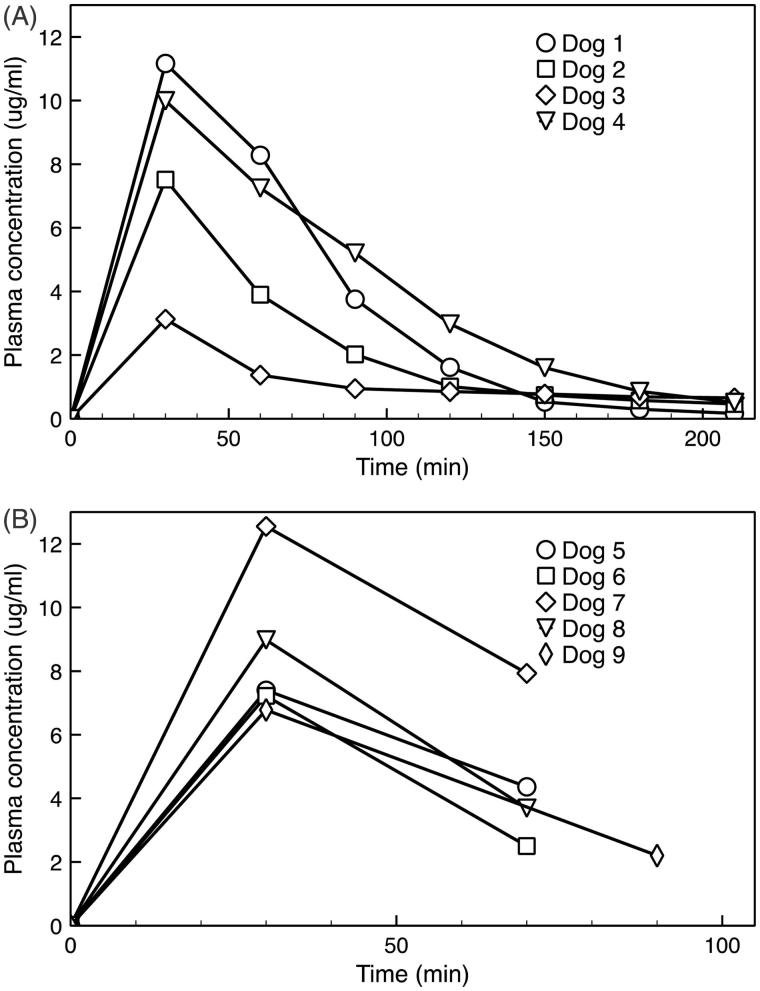
(A) Plasma PK for four animals in the nonsurvival study. Dog 3 had slightly higher body temperature (∼38 °C) during anesthesia than intended, explaining the considerably lower plasma drug concentration that likely resulted from premature systemic drug leakage from the TSL. (B) Plasma PK for five animals in the survival study, where only two plasma samples following drug infusion were obtained in each animal.

In acute dogs, plasma concentrations were measured by both HPLC and fluorometry methods with comparable results (*R*^2^ = 0.96). A peak plasma concentration of 8.0 ± 3.1 μg/g was observed at the end of drug infusion (Figure 5(A)). Initial plasma half-life was 45 ± 11 min, which was comparable to the 55 min half-life observed with a similar TSL-dox formulation in human patients (Wood et al., 2012). The plasma concentration was considerably lower in dog 3, likely due higher body temperature (∼38 °C) during and following infusion that lead to premature leakage of drug from TSL. For comparison, the body temperature of the remaining three dogs during and after infusion was ∼35–36 °C. The peak plasma concentration in the five survival dogs was 8.6 ± 2.4 μg/g, and thus comparable to the nonsurvival dogs (Figure 5(B)). The variation in plasma PK between dogs is likely due to biological variability in distribution and clearance, as similar variability was observed in a prior study with TSL-dox in canines (Hauck et al., 2006).

### Doxorubicin tissue uptake

**Figure 6.**
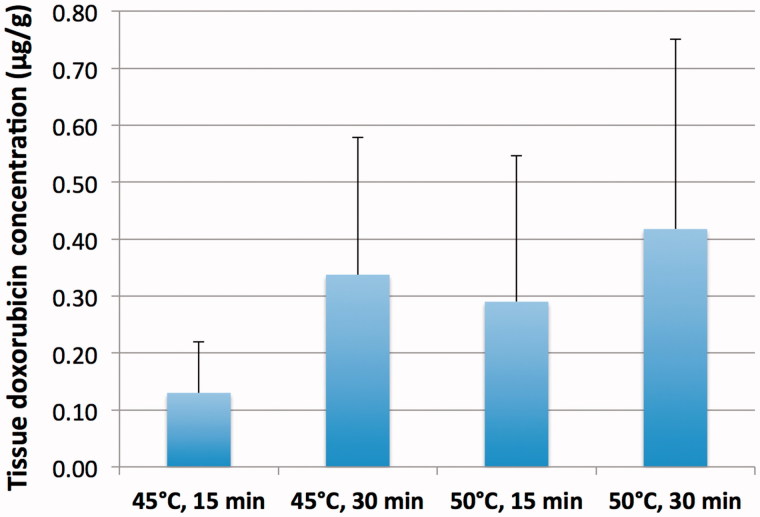
Tissue doxorubicin concentration separated by hyperthermia condition. There was a trend toward larger drug concentration at both higher temperatures and longer durations, but these did not reach statistical significance.

Tissue doxorubicin uptake ranged from 0.11 to 0.74 μg/g when detectable (Figure 6 and Table 2), and was only detected within 2 mm from the hyperthermia probe.

**Table 2.** Tissue doxorubicin concentrations of tissue exposed to hyperthermia.

Dog number	Probe temperature (°C)	Hyperthermia duration (min)	Sample weight (mg)	Tissue doxorubicin concentration (μg/g)
1	45	15	27	0.17
	45	30	29	0.55
	50	15	20	0.21
	50	30	45	0.15
2	45	30	47	0.34
	45	15	24	0.2
	50	15	32	0.12
	50	30	42	0.11
3	50	15	24	0.16
	50	30	40	0.67
	45	15	34	0
	45	30	34	0
4	50	30	26	0.74
	50	15	27	0.67
	45	30	36	0.46
	45	15	28	0.15

All of the control tissue samples (i.e. not exposed to heat) had undetectable doxorubicin concentration.

Analysis was done via HPLC and confirmed with fluorescence microscopy. Two samples demonstrated no delivery of doxorubicin in dog 3, which experienced systemic elevated temperature (∼38 °C) prior to the hyperthermia treatments. This elevated temperature likely resulted in systemic TSL drug leakage as also indicated by the rapid decline in plasma concentration in this animal (Figure 5), thereby diminishing drug available for delivery across a presumptively permeable BBB.

**Figure 7.**
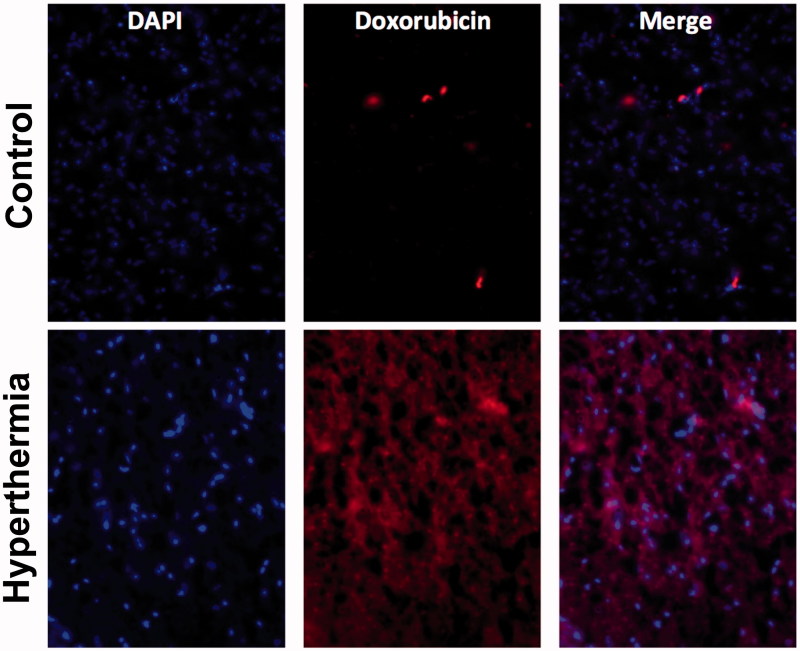
Fluorescence microscopy demonstrates localized doxorubicin delivery to tissue regions surrounding the hyperthermia probe (50 °C, 30 min).

Microscopic fluorescence images of the tissue samples were also attained (half of each brain sample was used for quantification, the other half reserved for imaging) and provided confirmation of the doxorubicin delivery (Figure 7). Those with strongest doxorubicin fluorescence signals were observed in samples treated with the most intense hyperthermia regimens.

### Statistics results

**Figure 8.**
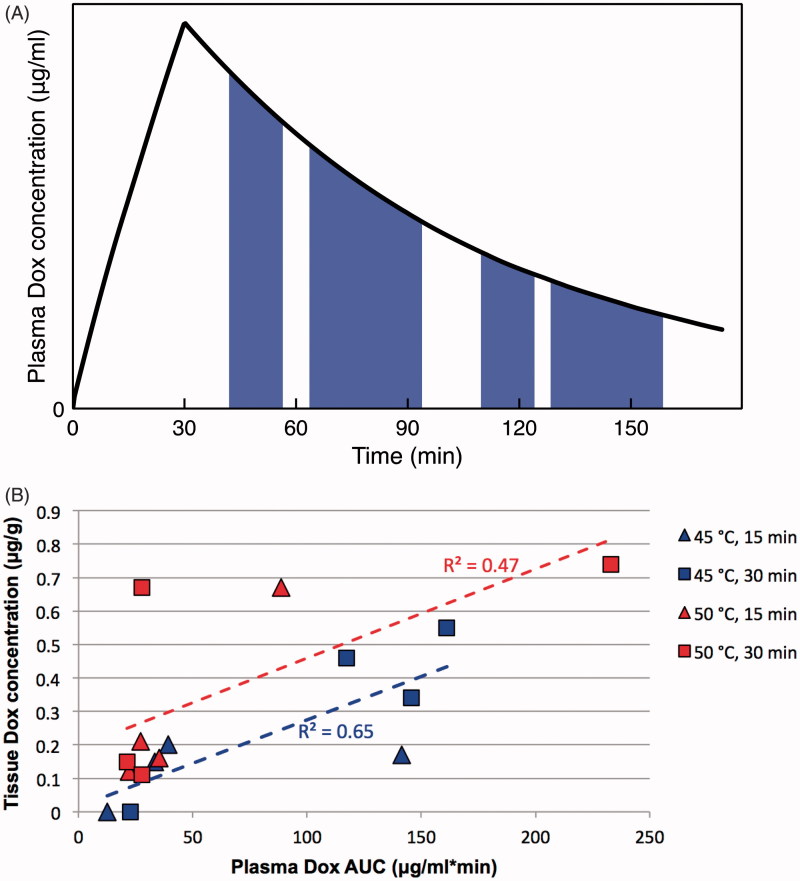
Plasma concentration area under the curve (AUC) predicts tissue drug uptake. (A) AUC was calculated based on plasma concentration measured in each particular animal, during the time when hyperthermia was applied. The four shaded regions represent the plasma AUC’s corresponding to the four hyperthermia applications in dog 1 (15, 30, 15, and 30 min, respectively; this sequence varied among animals per Table 2). (B) Plasma AUC calculated during hyperthermia correlates with tissue drug concentration. Each marker represents a tissue sample where drug concentration was measured, plotted against plasma AUC calculated when hyperthermia was applied for that particular sample and animal. Marker color indicates hyperthermia temperature of 45 °C (blue) or 50 °C (red); marker shape represents duration of 15 min (triangles) or 30 min (squares). Dashed lines represent linear fits for each of the two temperature data sets as used in the statistical model B. Plasma AUC was a statistically significant predictor (*p* = .003) of tissue drug concentration, and temperature trended toward statistical significance (*p* = .07).

Average tissue drug concentration for each condition is shown in Table 3. On average, a duration of 30 min resulted in a 0.17 μg/g higher concentration compared to 15 min, and probe temperature of 50 °C resulted in 0.12 μg/g higher concentration when compared to 45 °C (Tables 3 and 4); while these trends were as expected, neither of these fixed effects in Model A (linear mixed model with temperature, and duration as fixed effects) were statistically significant. In Model B (linear mixed model with temperature, and plasma AUC as fixed effects), plasma AUC was statistically significant (*p* = .003), and temperature trended toward statistical significance (*p* = .07) (Table 4). The negative log-likelihood was lower in Model B, indicating a better fit, and the simple *R*^2^ was higher. However, the likelihood ratio test comparing Models A and B resulted in *p* = .34, suggesting neither model was significantly better than the other. Given these model fit statistics, Model B using plasma AUC is preferable. Figure 8 visualizes the relationship between plasma AUC (during hyperthermia) and delivered drug.

**Table 3.** Average tissue concentration from regression analysis.

Least square mean concentration, μg/g (SE)	Duration (15 min)	Duration (30 min)
Temperature: 45 °C	0.15 (0.11)	0.32 (0.11)
Temperature: 50 °C	0.27 (0.11)	0.44 (0.11)

**Table 4.** Linear mixed effects regression results.

	Regression coefficient (SE)	*p* value	–Log likelihood^a^	*R*^2a^
Model A				
Duration	0.17 (0.11)	0.16	2.35	0.19
Temperature	0.12 (0.11)	0.30	–	–
Model B				
AUC	2.6e-3 (6.8e-4)	0.003	3.36	0.56
Temperature	0.18 (0.09)	0.07	–	–

a Random effect was removed from models.

### Pathology and histology

**Figure 9.**
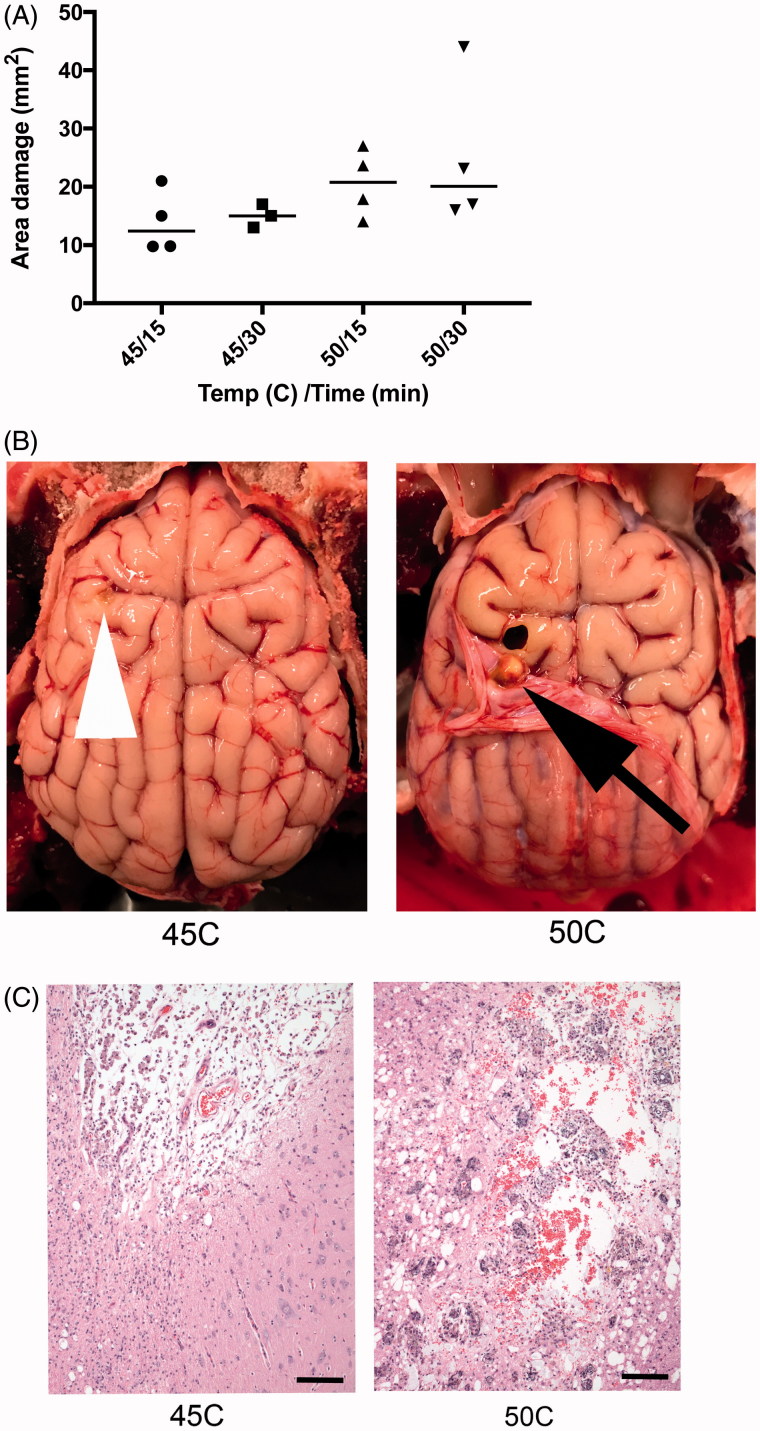
(A) Higher temperature and longer hyperthermia duration resulted in increased area of cerebral damage, measured from gross sectioned tissue in acute animals. (B) There is a defect in both brains, but to a lesser extent in animal exposed to 45 °C (tip of white arrow head). In animals exposed to 50 °C, there is a gross cavity within cerebral parenchyma and an associated expansion of the dura mater (black arrow). (C) Histology damage assessment. Both images are at 100× magnification and represent the ventral aspect of the probe insertion site. There is less damage to the surrounding parenchyma in the dog exposed to 45 °C (left) compared to 50 °C (right). Animals exposed to 50 °C had increased hemorrhage, vacuolization (clear spaces), and inflammatory response. Scale bar is 100 μm.

In acute animals, images and measurements of damage were obtained after gross sectioning of brain. Volume of damage was not possible to assess due to sectioning, but 2D area of damage was measured. Higher temperatures resulted in increased area of cerebral damage (Figure 9(A,B)).

Histologically, this trend remains. Sites receiving increased temperatures or for increased duration show a greater degree of histological abnormality (Figure 9(C)). Hyperthermia at 45 °C for 15 min resulted in minimal meningeal hemorrhage with minimal associated cerebral malacia. At the same temperature for 30 min, changes included increased neuronal damage. Damage incurred at 50 °C for 15 min consisted of focal neuronal necrosis, hemorrhage and edema of cerebral neuropil. When time was extended to 30 min, the area affected was larger, and there was increased neuronal necrosis. There were few instances where damage to neurons expanded beyond areas affected grossly, particularly in areas of greater neuronal susceptibility to damage (hippocampus).

In animals undergoing survival surgery, the gross lesions were consistent with those seen in the acute surgical animals, although there was the opportunity for healing. There were focal expansions (1 × 1 × 1 mm) of the dura mater corresponding to and invaginating into gross cerebral cysts. Histologically, animals undergoing the chronic procedure showed evidence of healing of the area of damage with abundant gitter cells, recent and remote hemorrhage, neovascularization, vacuolization, and astrocyte activation. The degree of histological damage was more significant in the dogs exposed to hyperthermia at 50 °C (30 min), compared to the one dog exposed to 45 °C (30 min).

### Tolerability of brain hyperthermia

Concern for neurologic toxicity of the hyperthermia regimen with localized doxorubicin spawned the short-term (6 weeks) survival study for the canines. Similarly, the first survival animal was treated more conservatively to limit potential complications (45 °C for 30 min). Only after a 1-week observational period following treatment of this first animal without adverse effects, we decided to employ the more aggressive hyperthermia protocol (50 °C temperature for 30 min) for the subsequent animals. No animal demonstrated significant or lasting neurological deficits after treatment as defined by thorough neurological examination and ability to perform activities of daily life (e.g. drinking, eating, urinating, defecating, walking, running, interacting with other dogs, and humans). One animal had the complication of a thermal skin burn on the sternum due to insufficient grounding of the equipment during treatment. Secondary intention healing of the burn in this animal was uncomplicated. Postoperatively, one other dog exhibited mild, nonprogressive right-sided proprioceptive deficits only during nonvisual-assisted hopping, and wheel barrow manipulations. Gait was normal except for mild internal rotation of the right thoracic limb. Activities of daily life were not impacted.

## Discussion

The BBB prevents or greatly limits delivery of the vast majority of drugs to the brain and thus is a primary reason why most therapeutic cancer agents are ineffective for brain tumors (Groothuis, 2000; Lesniak & Brem, 2004; van Tellingen et al., 2015). TSL are triggered drug delivery systems that release the contained drug in response to hyperthermia, typically >40 °C (Gaber et al., 1995; Needham et al., 2000; Lindner et al., 2004; Motamarry et al., 2017). Some more recent TSL formulations release very rapidly within seconds (Needham et al., 2000; Lindner et al., 2004). These TSLs take advantage of the intravascular triggered release concept, where drug is released while liposomes transit the vasculature of the heated tissue region (Gasselhuber et al., 2012; Manzoor et al., 2012; Kneidl et al., 2014; Motamarry et al., 2017). Since hyperthermia has been demonstrated to transiently open the BBB (Ikeda et al., 1994; Kakinuma et al., 1996; Kiyatkin & Sharma, 2009; Leuthardt et al., 2016), we hypothesized that the combination with fast-release TSL may be effective in delivering drugs across the BBB. In this paradigm, hyperthermia opens the BBB, and at the same time triggers intravascular release from TSL in the tissue regions where the BBB has been compromised, allowing for tissue drug uptake (Figure 1).

We employed TSL similar to the formulation initially proposed by Needham et al. (2000), and loaded with the chemotherapy doxorubicin as this drug is effective against glioma *in vitro* (Stan et al., 1999; Veringa et al., 2013; Sewing et al., 2017), but cannot cross the BBB. To elicit localized brain hyperthermia, we employed an interstitial needle probe based on radiofrequency heating, inserted through skull burr holes. Quantitative measurements of extracted brain tissue by HPLC demonstrated the ability to deliver potentially therapeutic doses of doxorubicin to brain tissue near the hyperthermia probe, with undetectable tissue concentrations in unheated brain regions (Table 2). Prior studies suggest that concentrations above 0.2–0.6 μg/g can provide a survival benefit in a rodent glioma model (Gong et al., 2011). Fluorescence microscopy of extracted tissue samples confirmed doxorubicin limited to the vicinity of the hyperthermia probe (Figure 7). Drug delivery was limited to a radius of 0–2 mm from the hyperthermia probe, with no detectable doxorubicin in tissue samples taken at greater than 2 mm distance or in control samples far from the probe. This demonstrates that the BBB permeability is enhanced in highly localized fashion depending on temperature. While we did not quantify the temperature required for BBB opening, prior studies in dogs suggests that a minimum of 41–43 °C is required (Ikeda et al., 1994; Kakinuma et al., 1996), while rodent studies indicate enhanced BBB permeability above ∼39 °C (Kiyatkin & Sharma, 2009).

While both temperature and time likely impact TSL-based drug delivery (Figure 6) (Rossmann et al., 2017), these effects did not reach significance in a linear mixed model due to low subject number. In a second statistical model, we considered the two parameters of temperature, and AUC of plasma drug concentration calculated during hyperthermia application (Figure 8(A)). In this second model, the effect of temperature approached significance (*p* = .07). Further, we did find a significant dependence (*p* = .003) of the amount of drug delivered based on AUC of plasma drug concentration calculated during hyperthermia (Figure 8). This observation demonstrates the importance of (1) adequate hyperthermia duration, and (2) application of hyperthermia while plasma concentration of encapsulated drug is high. The plasma concentration of encapsulated drug represents the amount available for release from TSL. During intravascular triggered release, TSL encapsulated drug continuously enters the heated region and is released as long as hyperthermia is present (Figure 1). I.e. the longer hyperthermia is applied, the more drug is released. Thus, the plasma AUC calculated during hyperthermia is a measure of the total amount of drug released within the capillaries of the heated tissue region, explaining the good correlation of this AUC with tissue drug uptake (Figure 8(B)). This ability of plasma AUC to predict tissue uptake has also been suggested by a prior computer modeling study (Rossmann et al., 2017). Our results further demonstrate the importance of having a TSL formulation that remains stable until the application of hyperthermia has been completed. For example, the low drug uptake (<0.2 μg/g) for the cluster of tissue samples in the left lower quadrant of Figure 8(B) can be explained by a low plasma concentration (and hence low plasma AUC) of encapsulated drug during application of hyperthermia.

We observed a lack of neurological toxicity in the survival animals. Admittedly, concern for neurotoxicity limited the magnitude of temperatures chosen to focus on potential neurotoxic effects of doxorubicin delivered to normal brain tissue. Our results demonstrate that localized delivery of doxorubicin appears to be safe.

While we demonstrated that hyperthermia could open the BBB, we also observed heat-induced damage near the heating probe (Figure 9). This suggests that accurate temperature control is required to avoid normal tissue toxicity due to hyperthermia while obtaining sufficient temperatures as required for BBB opening. Another potential danger is that drug uptake by endothelial cells causes vascular shutdown, which has been demonstrated in mouse tumors (Chen et al., 2004, 2008). While one of the studies suggests that vascular shutdown may be limited to tumors and not apply to normal tissues (Chen et al., 2004), this should be further examined in the normal brain in future studies. Damage present may also be due to the mechanical disruption of the cerebral cellular population or due to the neurotoxic effects of doxorubicin. The toxicity of doxorubicin is what makes it an effective chemotherapeutic agent, and why this approach to deliver it focally and precisely is so important.

Thermal therapies such as laser interstitial thermal therapy (LITT) or high intensity focused ultrasound (HIFU) are clinically used for treatment of brain disorders, including cancer (Wang et al., 2015; Leuthardt et al., 2016). These thermal therapies are typically coupled with MR thermometry, which allows noninvasive monitoring of tissue temperature (Rieke & Butts Pauly, 2008; Fahrenholtz et al., 2018), and may thus enable adequate temperature control to ensure temperatures are in the ideal range for BBB opening and localized drug delivery to the brain with TSL. In particular, HIFU may enable the delivery of chemotherapy to targeted brain regions in the clinics via a noninvasive hyperthermia modality.

Since it has been demonstrated that the BBB is compromised surrounding thermal lesions following LITT of brain cancer (Leuthardt et al., 2016), the combination of LITT with TSL in the clinic may enable drug delivery to the treatment zone margin to limit recurrences. This would be similar in concept to the combination of TSL with tumor ablation in the liver, where drug delivery to the margin around the ablation zone has been demonstrated (Swenson et al., 2015).

## Conclusions

We demonstrated that TSL combined with 15–30 min hyperthermia allows for localized delivery of potentially therapeutic doses of doxorubicin across the BBB. Survival studies suggest that this therapy is safe, with no or only limited normal tissue toxicity.
